# The role of imaging in the skeletal involvement of mucopolysaccharidoses

**DOI:** 10.1186/s13052-018-0556-z

**Published:** 2018-11-16

**Authors:** Vincenzo Spina, Domenico Barbuti, Alberto Gaeta, Stefano Palmucci, Ernesto Soscia, Marco Grimaldi, Antonio Leone, Renzo Manara, Gabriele Polonara

**Affiliations:** 1 Division of General and Emergency Radiology, Sant’Agostino-Estense Hospital, Modena, Italy; 2 Department of Diagnostic Imaging, Pediatric Hospital Bambin Gesù, Rome, Italy; 3 Radiology Unit, Pediatric Hospital Giovanni XXIII, Bari, Italy; 4 Radiodiagnostic and Radiotherapy Unit, University Hospital Policlinico-Vittorio Emanuele, Catania, Italy; 5 Bio-structures e Bio-imaging Institute, CNR, Naples, Italy; 6 Department of Diagnostic Imaging, Neuroradiology, Humanitas Clinical and Research Center, IRCCS-Rozzano, Milan, Italy; 7 Fondazione Policlinico Universitario Policlinico Gemelli IRCCS, Università Cattolica del Sacro Cuore, Rome, Italy; 8 Neuroradiology, Department of Medicine and Surgery, University of Salerno, Baronissi, Salerno, Italy; 9 Neuroradiology Unit, University Hospital “Ospedali Riuniti di Ancona”, Politecnica University of Marche, Ancona, Italy; 10 Radiology Unit, San Gerardo Hospital, Monza, Italy

**Keywords:** Mucopolysaccharidoses, Musculoskeletal involvement, Radiography, Ultrasound, Multidetector computed tomography, Magnetic resonance imaging

## Abstract

This article discusses the role of imaging modalities including radiography, multi-detector computed tomography, magnetic resonance imaging, and ultrasound in diagnosing and monitoring skeletal abnormalities in mucopolysaccharidoses (MPS). The advantages and disadvantages of these different imaging tools will be discussed, along with their feasibility in this class of patients. As the musculoskeletal involvement is common to all MPS and is one of the main reasons for seeking medical attention, an increased awareness among paediatricians, rheumatologists, orthopaedists, radiologists, and other musculoskeletal specialists on the possible spectrum of abnormalities observed could facilitate a timely diagnosis, an appropriate severity evaluation, and better management.

## Background

Mucopolysaccharidoses (MPS) are characterized by musculoskeletal involvement [1, 2]. The accumulation of partially degraded glycosaminoglycans (GAGs) in the lysosomes of connective tissue cells and chondrocytes is believed to be responsible for most of the musculoskeletal manifestations associated with the different forms of MPS [3]. To date, seven distinct clinical types (I to IV, VI, VII, and IX) and numerous subtypes of MPS have been described. Different residual enzymatic activity can result in different phenotypes of the same MPS type, from severe to attenuated [4, 5]. Skeletal and joint abnormalities vary widely both between and within each MPS type. The joint disease in MPS is progressive and characterized by the absence of significant clinical signs of inflammation. Joint stiffness and contracture can be present in all types of MPS except Morquio syndrome (MPS IV) which is characterized by joint hypermobility. Each MPS is associated with primary skeletal dysplasia that is referred to by the descriptive term dysostosis multiplex [4, 5]. The recent progress in early diagnosis and the existence of potentially effective therapies could have an immediate effect on the natural course of these chronic diseases. Since MPS have a wide variety of clinical presentations, diagnosis is often delayed and many children suffer for years with an unrecognized disease. More so than in the past, management of MPS patients requires a multidisciplinary approach because of the multiorgan nature of the disease. Imaging modalities play a key role in every phase of the management (diagnosis, treatment, and follow-up).

### What are the imaging modalities in the clinical practice of MPS patients? Why and when do we need to use them?

Imaging modalities that clinically used for the study of the musculoskeletal and visceral involvement of MPS are radiography, multidetector computed tomography (MDCT), magnetic resonance imaging (MRI), ultrasound with both color and power Doppler, and dual energy x-ray absorptiometry (DEXA) (Table 1).

**Table 1:** Musculoskeletal imaging modalities in mucopolysaccharidoses

	Axial	Appendicular	Articular
Standard radiography	+	+	+
MDCT	+	–	–
US (PW, CD)	–	–	+
MRI	+	+	+
DEXA	+	+	–

*CD* colour Doppler, *DEXA* dual energy x-ray absorptiometry, *MDCT* multidetector computed tomography, *MRI* magnetic resonance imaging, *PD* power Doppler, *US* ultrasound

These imaging modalities are used at different clinical phases of disease to: a) support the diagnosis of a suspected MPS and play a specific role in evaluating the severity and extent of dysostosis and joint involvement; b) monitor the chronic and progressive course of MPS; c) plan ad-hoc surgical procedures; and d) assess the impact of therapy.

Radiography is the immediate and informative first-line imaging modality to document gross skeletal abnormalities. Dysostosis multiplex is the term used to describe the group of radiographic changes characteristic of MPS. Malformation of the skull, chest, spine, pelvis, long bones, and hands are generally shared by several MPS types and can be revealed by radiography [4, 5]. This modality provides two-dimensional pictures of the skeleton and of the spine in particular, allowing the detection of scoliosis, kyphosis, craniocervical junction abnormalities, intervertebral instability, and spinal stenosis; furthermore, it gives a comprehensive evaluation of the severity and extent of dysostosis.

The skull might show an abnormal J-shaped sella turcica and a thickened diploic space. Gibbus deformities occur in nearly all children with severe forms of MPS such as Hurler syndrome (MPS I). The spine might present kyphosis as a result of poor bone growth in the anterosuperior aspect of lumbar vertebrae. Scoliosis has also been observed in MPS I, Hunter syndrome (MPS II), and Sanfilippo syndrome (MPS III), but it is rarely severe enough to require surgery. The craniocervical junction abnormalities, spinal stenosis, and intervertebral instability assessed during functional radiography can be the result of the process. Patients with MPS have an increased incidence of hypoplasia of the odontoid process, predisposing them to atlanto-axial instability.

**Fig. 1:**
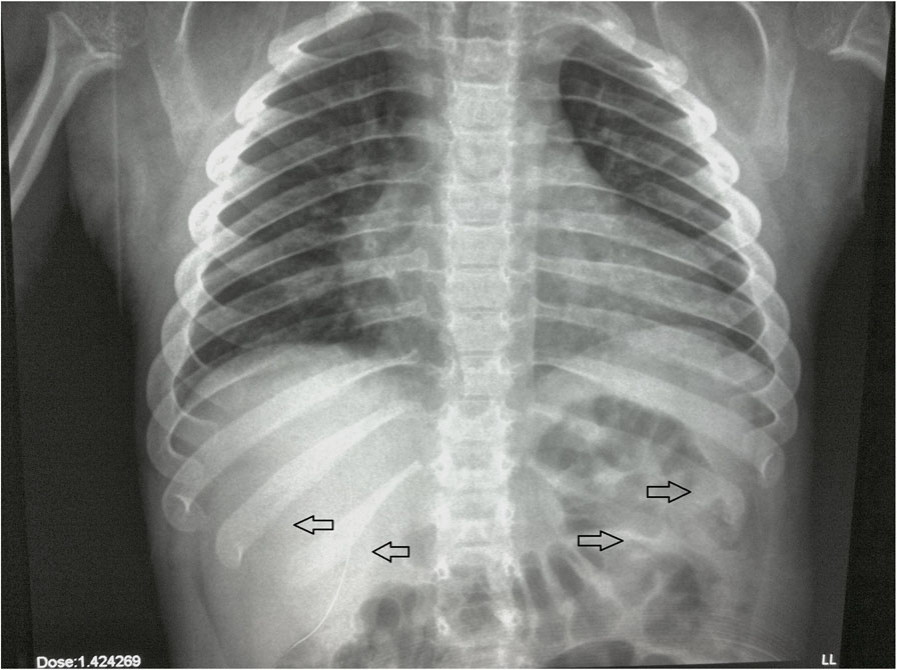
Typical picture of paddle-shape ribs in a patient with MPS IV (arrows)

**Fig. 2:**
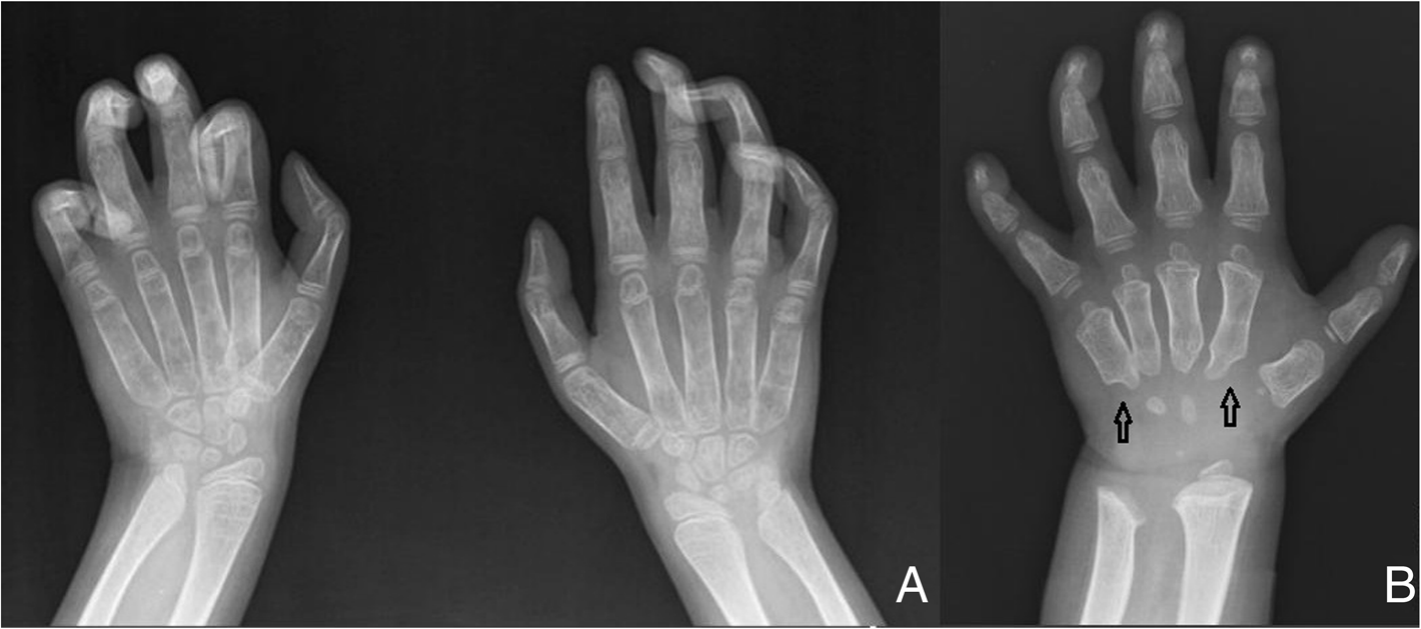
**a**. Claw hand and clinodactyly affecting II, III, IV and V left fingers and III, IV and V right fingers; Madelung deformity of the distal radius and ulna bilaterally. **b** Typical bullet-shaped phalanges and short metacarpals with proximal pointing (arrows) in MPS I- Hurler

**Fig. 3:**
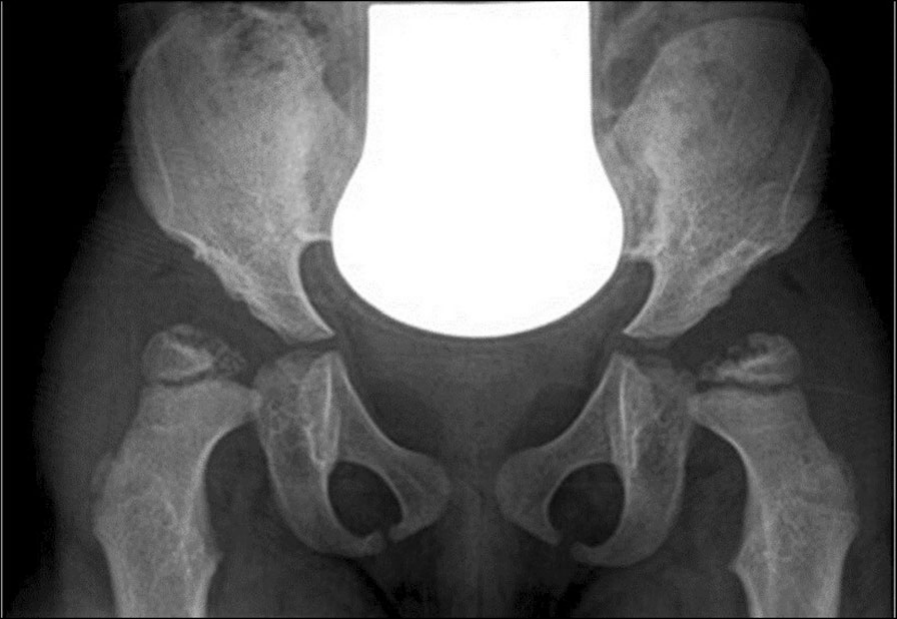
4-year-old with MPS VI. X-ray of the pelvis showing enlarged and receding acetabulum, underdeveloped femoral epiphysis, and acetabulum warped with coxa valga

The thorax might reveal paddle-shaped ribs, anteriorly widened and posteriorly tapered [6] (Fig. 1). Axial and peripheral skeletal imaging findings dominate the clinical radiological picture in Morquio (MPS IV) and Maroteaux-Lamy (MPS VI) syndromes. Among patients with MPS IV, a gibbus is generally reported in the first year of life, followed by a severe growth failure associated with short stature, short neck and trunk, genu valgum, hyperlordosis, scoliosis, ulna deviation, and broadening of the wrist [7]. Patients with MPS VI may present disproportionate dwarfism as the first symptom in the third year of life, even if coarse facial features, macroglossia, hepatosplenomegaly, and joint contractures are additional clinical-radiological findings [7]. Almost all forms of MPS show distortion of both hand and foot structures [4]. Carpal and tarsal bones are hypoplastic and irregularly shaped; the metacarpal bones are proximally pointed, shortened, and thickened (Fig. 2). The distal ulna and radius can be hypoplastic and are “V shaped”; this oblique deformity of the terminal part of both bones results in alteration of the carpal angle. In MPS, the long bones are often characterised by several alterations. Diaphysis are shortened and curved in the distal part; epiphyses are slightly hypoplastic and thinned cortically with osteoporosis [4, 5]. Notching of the proximal part of the humerus, a long and narrow aspect of the femoral neck, and hypoplasia of the lateral tibial hemiplate resulting in genu valgum are additional features. The most common radiological features in the pelvis are rounded iliac wings and inferior tapering of the ileum [8]. The alterations of the hip joint can lead to hip dysplasia because of the poor development of the acetabulum and the underdevelopment of the medial portion of the proximal femoral epiphysis (Fig. 3). Severe hip dysplasia is found in nearly all children with Hurler syndrome, but can also be found in the mildest form of MPS type 1 (Scheie syndrome). These alterations have not been shown to respond to medical therapy and for these children surgical reconstructions are often required [9].

MDCT generates three-dimensional images derived from a large series of two-dimensional x-ray images and allows the identification of more subtle bone anomalies, even if its power to image soft tissue is much less potent than MRI. When radiographic examinations are difficult to interpret, computed tomography (CT) can be useful to study the axial skeleton. MDCT has been applied for non-invasive imaging of the airway, including paediatric patients [10], because of its ability to produce a vast quantity of volumetric data in a reduced amount of time, its high resolution and, consequently, reliable multi-planar and three-dimensional reconstructions. The study of the upper airways is particularly important in MPS patients who need to undergo anaesthesia/sedation for both imaging diagnostic examinations and surgery [11]. In fact, patency of the upper airways is often compromised by the presence of several anatomical and functional abnormalities, such as deformities of the jaw, neck and chest, macroglossia, adenotonsillar hypertrophy, and thickening of laryngopharyngeal tissues [12]. In addition, dense mucus and structural tracheal abnormalities make anaesthesia a very risky procedure. According to data from the Hunter Outcome Survey [13], collected from 527 patients with MPS II, 83.7% of patients underwent surgery with a median of three operations each and with a median age at first operation of 2.6 years. Besides the anatomical malformation of the upper airways there is the concomitant presence of restrictive pulmonary disease in combination with cardiovascular manifestations, diaphragm hypomobility (due to the visceral organomegaly), and the risk of spinal cord compression which accounts for the increased mortality following anaesthesia. Typical anaesthetic problems include airway obstruction after induction or extubation, intubation difficulties or failure, possible emergency tracheostomy, and cardiovascular and cervical spine issues. The most frequently used imaging modality to study upper airways is fibroscopy, which is quite invasive and often difficult to perform in children. In a recent study [14] MDCT resulted in the useful preoperative evaluation of airways and significantly influenced the preoperative management plan, modifying the planned anaesthesiology approach in 21% of patients. A correlation between MDCT and fibroscopy has been recently observed in 35 MPS patients [15]; the authors reported tracheal morphology abnormalities in 50–60% of patients, with the highest severity in MPS types II and IV.

The use of MRI to investigate the joint involvement of lower limbs has been recently recommended for the evaluation of the progression of these diseases [16]. MRI is also useful to identify osteonecrosis of the proximal and distal femur [17], as well as to evaluate the articular cartilage status. It can also provide detailed information on the surrounding soft tissue and joint cartilage allowing, in some cases, an earlier detection of joint involvement; moreover, it allows the follow-up of a given therapy on a selected joint. This imaging modality is indicated to study the spine, to detect abnormalities of the skull base and craniocervical instability, and to image the brain. However, the presence of metal artefacts following surgery precludes high-quality MRI and, in these cases, CT might be preferred.

Radiography associated with ultrasound and MRI can be important in detecting joint dislocations. Ultrasound, in concert with colour and power Doppler, is a non-invasive tool that might be useful in the evaluation of joint involvement for facilitating the differential diagnosis of MPS with rheumatic diseases, follow-up, and assessment of efficacy of treatment [18].

DEXA is a frequently used tool to evaluate bone mineral density (BMD) because of its low radiation exposure and rapid scan time. Patients with MPS have an increased risk of poor bone mineralization due to malnutrition and reduction in physical activity caused by pain, poor health conditions, or exercise intolerance [19]. Using DEXA, osteopenia and osteoporosis were found in 31% and 15%, respectively, of MPS patients aged under 19 years not selected for skeletal involvement, while eight patients with MPS I, II, and IV showed some increased BMD values after receiving enzyme replacement therapy (ERT) for 1–7.4 years, resulting in values almost all within the normal range, particularly after correction for short stature [19]. These data suggest that DEXA can be a useful tool to study BMD and to identify patients with increased fracture-related mortality and morbidity risk [20]. The diagnostic role and reliability of bone ultrasonography, which is a safer non-x-ray technique with a potential role in paediatric patients, are still to be defined.

### What kind of information should the radiologist pass to the orthopaedic surgeon?

The radiologist should provide the orthopaedic surgeon with important information for surgical treatment and follow-up. Current therapies such as ERT and haematopoietic stem cell transplantation (HSCT) have little beneficial impact on the progression of skeletal deformities. The severity of skeletal involvement can vary among and within various MPS disorders and often requires surgical intervention [21].

All patients should have a single baseline study at initial evaluation. The first-line imaging modality for the assessment of musculoskeletal abnormalities is radiography (Table 2).

**Table 2:** Skeletal imaging survey in patients with mucopolysaccharidoses

	Baseline	Follow-up
Cervical spine	A-P, L-L radiographs standing upright MRI	Yearly^a^
Thoracolumbar spine	A-P, L-L radiographs standing upright MRI	Yearly^a^
Hips/pelvis	A-P radiograph	Yearly^a^
Lower extremities	A-P radiograph standing upright	Yearly^a^
Forearms	A-P radiograph	
Hands	A-P radiograph	
Feet	A-P radiograph	

*A-P* anteroposterior, *L-L* lateral-lateral, *MRI* magnetic resonance imaging

^a^Yearly radiography frequency depends on the type of patient clinical manifestation and might be appropriate only for those patients presenting significant kyphosis and/or suffering from spinal pain; MRI is preferred for those patients with neurological problems as it allows better imaging of the spine and a related spinal cord compression

Radiography of the spine and hips should be performed on a single-scan long cassette (36 in.) that includes the entire thoracolumbar spine as well as the pelvis, if possible [21]. Radiographic examinations are made in the standing or sitting position with anteroposterior and lateral views, and deformities, such as thoracolumbar kyphosis or scoliosis, are assessed using the Cobb method. It is very important to measure a Cobb angle on the lateral view also across the focal deformity.

Cervical instability should be evaluated using functional radiography in the sagittal plane, achieved either in flexion or extension, with the close supervision of a clinician. If radiography is difficult to interpret, flexion/extension CT scans can be helpful. MRI of the cervical spine is particularly useful with regard to the craniocervical junction [22], as well as MRI evaluation of the thoracolumbar spine performed in patients with evident deformity, or development of neurological changes of the lower extremity.

The anteroposterior view of the pelvis, especially in the upright position, is very important for the evaluation of hip dysplasia. Depending on the age of the patient, angular values are calculated to assess the degree of hip dysplasia (acetabular index, Sharp’s acetabular angle, the centre-edge angle of Wiberg). Sharp’s acetabular angle is considered normal if it is < 39°; the acetabular index is considered normal if it is < 20°; and the centre-edge angle is considered normal if it is > 20° [23–25].

**Fig. 4:**
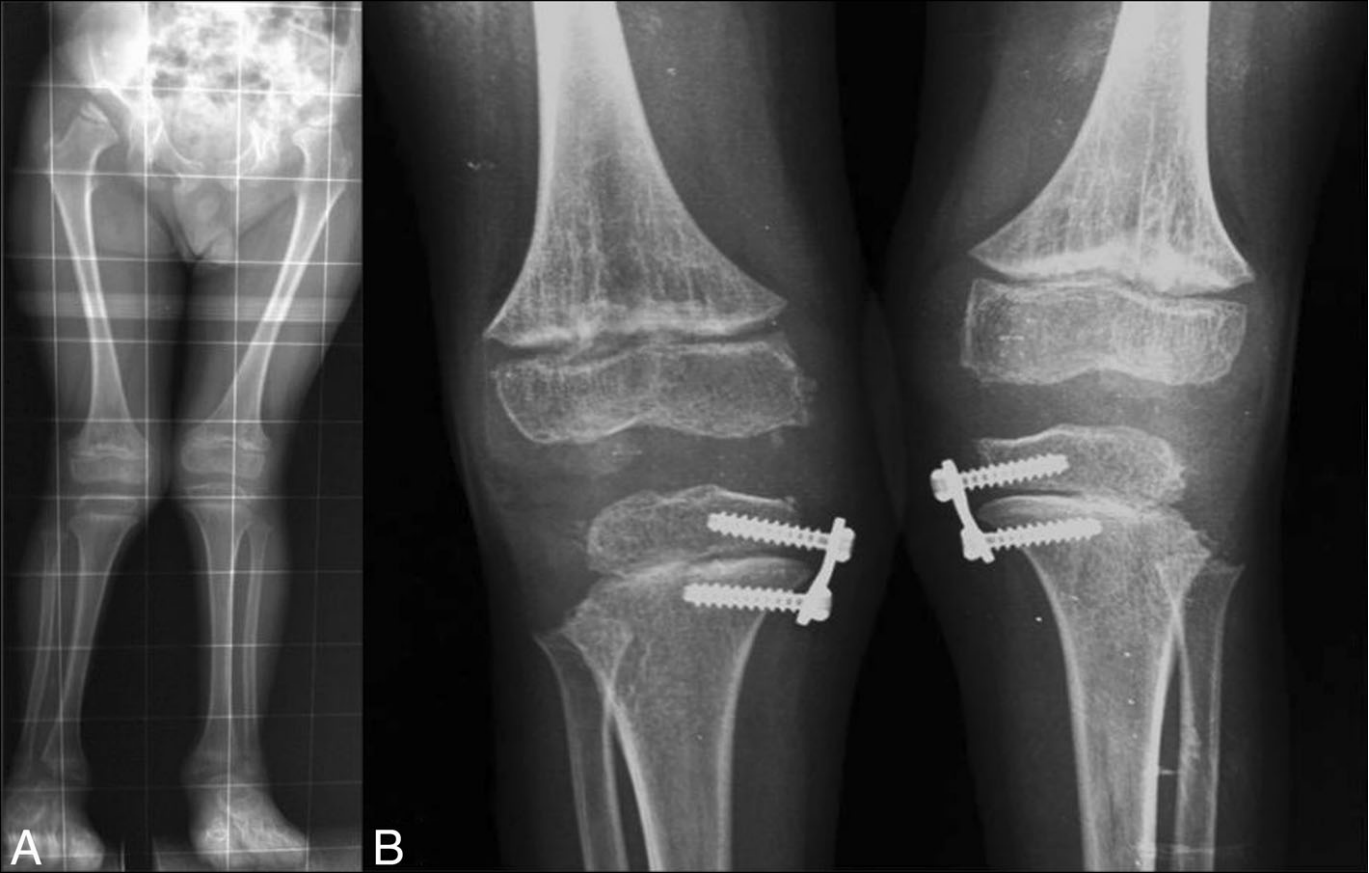
**a**. Bilateral genu valgum, more marked in the left knee, with severe acetabular hypoplasia in a patient with mucopolysaccharidoses I-H. **b** Same patient after surgical correction

Lower extremity alignment is best evaluated with a single anteroposterior view of both extremities in the standing position on a single cassette **(**Fig. 4**)**. In MPS, the typical deformity is genu valgum.

The follow-up of musculoskeletal lesions, performed with different imaging modalities, plays a key role in the evaluation of the progression of the pathology (Table 2). Radiography still represents the reference standard for monitoring the clinical course of musculoskeletal alterations; furthermore, it is useful for monitoring the therapeutic response in patients undergoing HSCT and ERT, even if no consensus exists on the frequency of the follow-up schedule (1-, 2-, or 3-year follow-up) [10]. In addition, the follow-up of spine lesions requires MRI to detect relapse and progression of the craniocervical stenosis along with progression of deformity of the bones.

When medical therapy alone is not enough, the skeletal involvement may require multiple orthopaedic surgeries with postoperative monitoring of the results and exclusion of orthopaedic complications. In these cases, radiography plays a key role. Surgery places a huge burden on MPS patients with severe somatic impairment. Approximately 75% of patients enrolled in an MPS I register were reported to undergo at least one surgical procedure, with an average of 3–4 surgeries per patient; specifically, by the ages of 1.5, 4, and 10 years, ≥ 2 surgeries were reported by 22%, 44%, and 54% of patients, respectively, the most common ones being for rupture/contracture of tendons, trigger finger and carpal tunnel, followed by hip, lower extremities, and spine interventions [26]. The results of the Morquio A clinical assessment program [27] have recently reported that almost 75% of MPS IVA patients under 12 years of age and more than 95% of patients over 12 years of age required surgical or medical interventions. Hip dysplasia is generally associated with acetabular hypoplasia and incomplete development of the medial portion of the proximal epiphysis. All these conditions will eventually lead to joint luxation. Hip dysplasia, when already present, has not been shown to respond to HSCT or ERT and most MPS patients after HSCT will eventually require corrective surgery. Hip surgery is not recommended for MPS III and IV [28] due to the possible development of femoral head osteonecrosis and collapse.

### What are the pros and cons of the imaging modalities in the management of MPS patients?

Beside the well-known safety and the economic issues related to the different types of imaging modalities, there are some important feasibility issues that should guide the use of one modality over the other. Patients with MPS are considered the most difficult category to study for several reasons. One of these is the fact that many of these patients are paediatric subjects who are intellectually impaired and cannot cooperate. In addition, most of these patients have walking problems and several contractures, thus making execution of the different examinations difficult both for postural reasons and for the patients inability to maintain the same position for a long time. The study of spinal stenosis requiring sagittal images might be difficult to realise because of the presence of scoliosis or kyphosis. While radiography is instrumental for a baseline assessment of dysostosis [5], no internationally recognized criteria for the evaluation of therapeutic radiological/imaging findings in MPS exist. Quantification of minor skeletal changes in dysostosis multiplex is a major challenge if we consider the great variety in bone alterations among individuals with MPS. The availability of a reproducible scoring system for an objective assessment would be very useful for the basal evaluation, during the follow-up, and for the appraisal of the response to therapy. Standardized assessment of radiological findings could provide insight into the natural course of bone disease in the different types of MPS. On the other hand, the regular use of radiography to monitor the progression of the disease and the impact of the therapy has to be balanced against the side effect of x-rays, considering that most patients, when first diagnosed, are just a few years old [4]. In our experience, considering the series of radiographs to be performed at the initial evaluation (as suggested by Muenzer et al. [29]) and the average relative effective dose (mSv) of a single examination, the overall effective dose for a 2-year-old child (height 80 cm, weight 11 kg) corresponds to 0.28 mSv and it increases to 0.60 mSv for a 10-year-old boy (height 140 cm, weight 32 kg). These values, compared with natural radiation exposure (2.4 mSv), are approximately equivalent to 1 and 3 months of exposure to background radiation, respectively, increasing the stochastic radio-induced risk. If we refer to the effective dose of posteroanterior chest x-ray (0.01 mSv in both ages), the effective dose for a plain film of the full column (0.14 mSv for a 2-year-old child and 0.43 mSv for a 10-year-old boy) basically corresponds to the effective dose for 14 and 43 chest x-ray examinations, respectively (Table 3).

**Table 3:** Skeletal imaging survey: dose exposure

	Effective dose (mSv)	
Radiographic examination	2-year-old child (height 80 cm, weight 11 kg)	10-year-old boy (height 140 cm, weight 32 kg)
A-P/L-L skull	0.05	0.08
A-P/L-L thoracolumbar spine	0.14	0.43
A-P lower extremities	0.01	0.03
A-P hips/pelvis	0.05	0.01
L-L cervical spine	0.02	0.04
P-A thorax	0.01	0.01
A-P forearms, hands, feet	0	0.001
Total	0.28^a^	0.60^b^

*A-P* anteroposterior, *L-L* lateral-lateral, *P-A* posteroanterior

^a^Corresponding to 1 month of background radiation dose, equivalent to twenty-eight posteroanterior thorax examinations

^b^Corresponding to 3 months of background radiation dose, equivalent to sixty posteroanterior thorax examinations

The recent development of new low-dose and ultra-low-dose CT technologies can minimize the exposure of patients to x-rays [30]. These new techniques allow the analysis of the cranial suture, brain, middle and inner ear, and cervical spine in a single scan over a time frame of a few seconds (from 2 to 10 s). All this can be obtained with the use of reduced x-rays (less than 70–75%) and with an even greater spatial resolution and contrast imaging [31]. A quick examination is very important in all cases of severe malformation or poor collaboration from the patient. MRI has a predominant role in the study of the spine, the brain, the soft tissues, and small joints, providing very detailed information on the soft tissues and joint cartilage [4]. MRI is efficient as it does not use x-rays; however, the presence of metal artefacts precludes high-quality MRI after several surgical procedures and, in these cases, CT is preferred. In addition, this technique requires patients to be still for a long time and this is not always possible in MPS patients. Finally, MRI is generally much more expensive than radiography and CT. If we consider that most patients are paediatric, it would be extremely useful to have MRI open machines with different designs more suitable for patient positioning. Indeed, the available orthopaedic reels to be used with the current machines are often very stiff and poorly suited to cope with the capabilities of patients. Again, a more common use of the upright MRI could be beneficial for the patients.

## Conclusions

MPS are chronic, disabling, and progressive diseases with both cognitive and physical alterations. Since the introduction of HSCT and ERT, the natural history of MPS has changed, especially if a therapeutic regimen is begun early in the course of the disease. A positive effect on growth velocity, for example, is more evident in patients who started ERT treatment before the age of 10 years. As outlined in this article, radiography, CT, and MRI play a key role in diagnosing MPS but proper imaging evaluation is also crucial in guiding appropriate treatment and follow-up. However, a multidisciplinary approach (by radiologists and clinicians) and the correlation of clinical and imaging findings are required.

## Keypoints

- Skeletal manifestations of MPS are common
- Appropriate imaging modalities have a key role to play in the management of MPS in every phase of the clinical course
- Regular monitoring of skeletal disease with radiography, CT, and MRI is recommended in MPS
- Radiologists should provide important information for sedation, surgical treatment, and follow-up to the anaesthesiologist and the orthopaedic surgeon

## Abbreviations

BMD: Bone mineral density
CT: Computed tomography
DEXA: Dual energy x-ray absorptiometry
ERT: Enzyme replacement therapy
HSCT: Haematopoietic stem cell transplantation
MDCT: Multidetector computed tomography
MPS: Mucopolysaccharidosi(e)s
MRI: Magnetic resonance imaging
